# Assessing the Effectiveness of Automatic Speech Recognition Technology in Emergency Medicine Settings: A Comparative Study of Four AI-powered Engines

**DOI:** 10.21203/rs.3.rs-4727659/v1

**Published:** 2024-08-17

**Authors:** Xiao Luo, Le Zhou, Kathleen Adelgais, Zhan Zhang

## Abstract

Purpose Cutting-edge automatic speech recognition (ASR) technology holds significant promise in transcribing and recognizing medical information during patient encounters, thereby enabling automatic and real-time clinical documentation, which could significantly alleviate care clinicians’ burdens. Nevertheless, the performance of current-generation ASR technology in analyzing conversations in noisy and dynamic medical settings, such as prehospital or Emergency Medical Services (EMS), lacks sufficient validation. This study explores the current technological limitations and future potential of deploying ASR technology for clinical documentation in fast-paced and noisy medical settings such as EMS. Methods In this study, we evaluated four ASR engines, including Google Speech-to-Text Clinical Conversation, OpenAI Speech-to-Text, Amazon Transcribe Medical, and Azure Speech-to-Text engine. The empirical data used for evaluation were 40 EMS simulation recordings. The transcribed texts were analyzed for accuracy against 23 Electronic Health Records (EHR) categories of EMS. The common types of errors in transcription were also analyzed. Results Among all four ASR engines, Google Speech-to-Text Clinical Conversation performed the best. Among all EHR categories, better performance was observed in categories “mental state” (F1 = 1.0), “allergies” (F1 = 0.917), “past medical history” (F1 = 0.804), “electrolytes” (F1 = 1.0), and “blood glucose level” (F1 = 0.813). However, all four ASR engines demonstrated low performance in transcribing certain critical categories, such as “treatment” (F1 = 0.650) and “medication” (F1 = 0.577). Conclusion Current ASR solutions fall short in fully automating the clinical documentation in EMS setting. Our findings highlight the need for further improvement and development of automated clinical documentation technology to improve recognition accuracy in time-critical and dynamic medical settings.

### 1. Introduction

Clinical documentation is a time-consuming and challenging task. Electronic health records (EHRs) have been increasingly adopted to streamline this process. However, research has shown that the introduction of EHR systems has led to increased clinician burnout [1], decreased time spent on patient care [2], and lower patient satisfaction [3]. The unintended consequences of using EHRs are exacerbated in more time-critical and dynamic settings, such as emergency medicine, because the documentation task in such settings is performed during a short and highly intense process, posing significant challenges in the timely and accurate creation of patient medical records, and thus, leading to incomplete, delayed, or erroneous documentation [4–6].

Given these challenges in the effective use of EHR in various medical settings, research has proposed using automatic speech recognition (ASR) technologies, empowered by natural language processing (NLP) techniques, to automate (part of) clinical documentation [7]. These technologies record and process conversations during patient-provider encounters to extract and summarize relevant clinical information. The processed information could, for instance, be used to populate some EHR fields, create billing codes, and generate decision and diagnostic support.

Some notable and advanced ASR tools are offered by companies such as Google [8], Nuance [9], and Amazon [10]. A few studies have assessed their performance in recognizing clinically meaningful information from patient-provider conversations [7, 11]. However, these prior studies were mainly conducted in quieter medical settings, such as patient exam rooms and the performance of ASR technology is understudied in more dynamic and noisier clinical settings. Additionally, prior work mainly investigated one-on-one interactions, such as between physician and patient during clinical encounters. The ability of these commercially available ASR tools to recognize medical information in non-linear, fragmented conversations involving multiple people such as occurs in in emergency care has not been investigated.

To address these remaining questions, our work uses Emergency Medical Services (EMS) or prehospital care as a study context, as it presents a perfect example of dynamic and noisy medical settings with many people involved (e.g., multiple care clinicians, patients, bystanders, family members, etc.). During prehospital care, EMS clinicians are dispatched to the field to provide emergency care, with the goal of quickly stabilizing a patient's urgent health condition and transporting the patient to the nearest or most appropriate care facility. Given the dynamic, hands-on, and time-critical nature of prehospital care, real-time clinical documentation using EHR is often impossible [12, 13]. These characteristics present a great opportunity for ASR technology to facilitate and automate EHR documentation in prehospital care.

In this study, we report an empirical assessment of how four commercially available ASR engines perform in transcribing and recognizing medical information from 40 audio recordings of high-fidelity simulations of prehospital care with teams of EMS clinicians. We aim to address the following research questions through this work: 1) How effectively do commercially available ASR engines transcribe and recognize clinically relevant information in dynamic and noisy emergency care environments such as in the prehospital setting? 2) What are the common types of transcription errors made by ASR engines in prehospital settings? Answering these research questions can inform the further development and improvement of ASR tools in supporting real-time clinical documentation in dynamic and noisy medical settings.

### 2. Methods

## 2.1 Automatic speech recognition (ASR) engines evaluated

This study focuses on evaluating the potential of leveraging ASR engines to facilitate clinical documentation in emergency care settings, such as EMS, which are often noisy, dynamic, and involve multiple parties in the care process, as opposed to quiet care settings such as medical exam rooms. Based on our research and literature review, Google Cloud Speech-to-Text [14] with the medical conversations model, OpenAI automatic speech recognition model Whisper [15], Amazon transcribe medical with conversation model [10], and Microsoft Azure Speech-to-Text [16] are the four most advanced ASR engines that are publicly available for evaluation. We refer to these four ASR engines in the following content as "Google ASR", "Amazon ASR", "OpenAI ASR", and "Azure ASR", respectively.

## 2.2 Evaluation dataset

The assessment data were generated from 40 audio recordings of high-fidelity EMS simulations, which were conducted in a mobile simulation lab resembling the back of an ambulance. The simulation scenarios varied by clinical conditions and required treatments, including a 15-month-old with seizure, a 1-month-old with hypoglycemia, and a 4-year-old with clonidine ingestion. These simulations replicate the dynamic prehospital environment, including background noise and overlapping speech from multiple speakers. The participants of the simulations were EMS clinicians who staff either an ambulance or a fire truck in their daily roles. The simulation recordings were transcribed by professional transcriptionists as part of a project examining EMS workflow [17, 18]. These transcripts are used as ground truth for ASR engine evaluation. The Institutional Review Board of Pace University thoroughly reviewed the study protocol and determined it to fall under the category of nonhuman subjects' research.

## 2.3 Annotation process for classifying transcription into EMS EHR fields

Given that the ultimate goal of our work is to determine how to leverage ASR engines to automate clinical documentation in EMS, the main aim of this assessment is to compare the ability of different AI-powered ASR engines to accurately transcribe content associated with specific structured fields within EMS EHR. Examples of such structured fields include "age," "gender," "past medical history," "allergies," and others. We used a multi-step process to annotate the transcripts.

First, based on the examination of two EMS EHR systems' structures as well as NEMSIS data structure [19], we listed all the typical structured fields in the EMS EHR systems and categorized them into five high-level categories: "ePatient", "eHistory", "eVital", "eSituation", and "eMedication" (Table 1). Then, two annotators (ZZ and LZ) independently annotated six transcripts from three different scenarios (two transcripts for each scenario) by marking content that belongs to these structured fields. In this step, we calculated the inter-rater agreement using Cohen's Kappa coefficient; two annotators achieved an "almost perfect agreement" on the mapping between content and EHR fields (kappa value is 0.80). All disagreements between the annotators were resolved in meetings that involved a third annotator (XL).

Afterwards, a data dictionary, as shown in Table 1, was created, which includes five high-level categories along with the corresponding EMS EHR fields under each category, as well as annotated examples. Finally, one annotator (LZ) annotated the rest transcripts based on the data dictionary, while another annotator (ZZ) reviewed all the annotations to ensure correctness and consistency of annotation across all 40 transcripts. It is worth noting that the annotation process was iterative and collaborative, involving back-and-forth correction of previous annotations.

Table 1
Categories and fields of data annotation with exemplars

| Category | EMS EHR fields | Example |
|---|---|---|
| ePatient | Gender | male |
| | Age | 15 month old |
| | Family information | has 3 year old brother |
| eSituation | Complaint/Symptoms | vomiting, diarrhea |
| | Lung sounds | clear |
| | Mental state | fussy |
| | Trauma | no signs of trauma |
| | Airway | clear |
| eHistory | Allergies | never had any allergies |
| | Past medical history | ADHD |
| eVital | AVPU (Alert, Verbal, Pain, Unresponsive) | only responds to painful stimulus |
| | BGL (Blood glucose level) | 100 |
| | BP (Blood pressure) | 58 over 36 |
| | Capillary Refill Time | 6 seconds |
| | ECG (Heart rate) | 190 |
| | Electrolytes | potassium |
| | Pupils | a little dilated and slow to respond |
| | Pulse | 190 |
| | RESP (Respiration rate) | 22 to 26 |
| | SPO2 (Oxygen saturation) | 100% |
| | Temperature | 98.4 |
| eMedication | Medication | D10 20 milliliters |
| | Treatment | put on mask to help breathing |

### 2.4 Automated content extraction from ASR transcribed text for performance evaluation

To determine the ASR engine's ability in transcribing clinically relevant information associated with structured fields in the EMS EHR, an automated content extraction framework is designed, shown as Fig. 1. This extraction framework aims to identify transcribed text that aligns with the annotated ground truth transcription. First, the timestamp of the ground truth transcription that contains the annotated content belongs to the EHR fields is used to locate the searching span of the text analysis. For example, if the ground truth transcription contains annotated age information, based on the timestamp of the transcription, we located the initial timestamp of the ASR transcribed data. From the initial timestamp, we included five ASR transcribed sentences before and after that timestamp for the text analysis. Then, a sliding window of length $n$ that matches the number of words in the annotated content is used to generate candidate content for extraction from the ASR transcribed sentences for similarity measurements. The sliding window does not cross punctuation tokens. For example, to match annotated age “one month old” in the ground truth transcription, a sliding window of length 3 can be applied to text “All right, so this is Susie, one month old.” to generate the following candidates “so this is”, “this is Susie”, “one month old”.

Two NLP similarity metrics were employed in this study: (1) Levenshtein distance similarity [20], which is based on fuzzy string matching [21], and (2) embedding-based semantic similarity, which is based on cosine similarity calculated using the large language model-based embeddings. These metrics were utilized to identify the most suitable matches in the ASR transcribed data compared to the annotated ground truth transcriptions. The Levenshtein distance serves as a metric for quantifying the dissimilarity between two sequences of words. This metric calculates the minimum number of edits required to transform one word sequence into another. The permissible edits include inserting, deleting, or substituting different letters or words. The FuzzyWuzzy ratio function [22] was used to estimate the Levenshtein distance similarity ratio between a candidate and annotated ground truth. To calculate the semantic similarity between the candidate and annotated ground truth, we first used BERT-based sentence transformer [23] to convert candidates and ground truth into embeddings. Then, cosine similarity is calculated to measure the similarity between them. The candidate demonstrating the highest Levenshtein distance similarity and cosine similarity was chosen as the best match and utilized for quantitative evaluation.

### 2.5 ASR performance evaluation methods

Two performance evaluation methods were employed to evaluate the extracted content against the annotated ground truth.

##### Named Entity Recognition (NER)-based evaluation metrics

After the matching content is extracted, the traditional evaluation metrics that are applied for NER tasks were used for evaluation [24–26]. All extracted content is converted into words. Then, the word-based precision (P), recall (R), and F1 are calculated for each category of the structured fields.

##### Semantic similarity-based accuracy measurement

For the semantic evaluation, cosine similarity between the embeddings of the annotated ground truth and the best matching candidate is calculated. If the cosine similarity is above the threshold 0.8, it is a correct match. The calculated accuracy for each category of the structured fields is based on the frequency of the correct match.

Table 2
Statistics of the annotated transcription data.

| Category | EMS EHR fields | Total # occurrences | % of recordings | Max # occurrences in each recording | Average length by words |
|---|---|---|---|---|---|
| ePatient | Gender | 26 | 62.5% | 2 | 1.00 |
| | Age | 92 | 100% | 5 | 2.47 |
| | Family information | 22 | 30% | 4 | 3.59 |
| eSituation | Complaint/symptoms | 379 | 100% | 22 | 2.88 |
| | Lung sounds | 37 | 47.5% | 5 | 2.35 |
| | Mental state | 1 | 2.5% | 1 | 1.00 |
| | Trauma | 27 | 30% | 5 | 3.89 |
| | Airway | 14 | 25% | 2 | 3.07 |
| eHistory | Allergies | 21 | 42.5% | 2 | 2.38 |
| | Past medical history | 190 | 92.5% | 15 | 2.77 |
| eVital | AVPU | 105 | 85% | 7 | 2.87 |
| | BGL | 68 | 80% | 4 | 1.38 |
| | BP | 158 | 92.5% | 12 | 2.61 |
| | Capillary refill time | 15 | 27.5% | 4 | 2.13 |
| | ECG | 108 | 87.5% | 11 | 1.73 |
| | Electrolytes | 3 | 2.5% | 3 | 1.00 |
| | Pupils | 11 | 15% | 4 | 2.54 |
| | Pulse | 69 | 65% | 7 | 1.84 |
| | RESP | 119 | 87.5% | 11 | 2.81 |
| | SPO2 | 69 | 72.5% | 8 | 1.55 |
| | Temperature | 37 | 50% | 4 | 1.86 |
| eMedication | Medication | 457 | 100% | 26 | 2.64 |
| | Treatment | 417 | 97.5% | 24 | 2.42 |

### 3. Results

## 3.1 Descriptive statistics of the evaluation data

The ground truth transcription of the 40 audio recordings each contained 744 to 1957 words (mean: 1340.5, Std: 297.57). The number of speakers in each recording ranged from 4 to 8 (mean: 6, Std: 1.02). The duration of the reenacted audio recordings was between 528 and 928 minutes (mean: 652.5, SD: 86.58).

The detailed statistics of the annotated data can be found in Table 2. One interesting observation is that the total number of occurrences in each EHR field varies. The most frequent ones are those belongings to EHR fields “complain/symptoms”, “medication”, and “treatment”, whereas the less frequent ones are “mental state” and “electrolytes”. Some categories occur in more than 87.5% of the recordings, such as “age”, “complain/symptoms”, “past medical history”, “BP”, “ECG”, “RESP”, “medication”, and “medication equipment”. Another interesting observation is that the average length of the annotated content in most fields is no more than three words. The category “trauma” has the longest average description, with 3.89 words, possibly because it often has detailed descriptions about the patient’s situation after the examination.

### 3.2 ASR performance evaluation and comparison

The performance of the four ASR engines in recognizing the content in EMS EHR categories is reported in Table 3. The results show that Google ASR has a higher overall average performance than the other three ASR engines. However, comparing to Google ASR, the other three ASR engines perform slightly better in transcribing information for certain categories, such as “gender”, “age”, “family information”, “lung sounds”, “capillary refill time”, “complaint/symptoms”, “trauma”, “airway”, and “pupils”. Amazon ASR and Azure ASR only performed significantly better than Google ASR and OpenAI ASR in transcribing “gender” information. Upon investigation, we found that “male” was often transcribed as “mail” by Google ASR and OpenAI ASR. All ASR engines have great performance on transcribing information about “mental state” and “electrolytes”. This could be because there are fewer instances in the evaluation data, and the descriptions of these two categories are relatively short. OpenAI ASR demonstrates consistently higher performance in categories such as “family information,” “trauma,” and “airway,” which typically have an average length of more than three words.

Table 3
**Performance comparison of the ASR using NER-based metrics**. *Note*: The numbers in bold highlight the best performance of each category.

| EMS EHR fields | Google ASR | | | OpenAI ASR | | | Amazon ASR | | | Azure ASR | | |
|---|---|---|---|---|---|---|---|---|---|---|---|---|
| | F1 | R | P | F1 | R | P | F1 | R | P | F1 | R | P |
| Gender | 0.654 | 0.654 | 0.654 | 0.731 | 0.731 | 0.731 | **1.000** | **1.000** | **1.000** | 0.962 | 0.962 | 0.962 |
| Age | 0.737 | 0.737 | 0.737 | 0.522 | 0.522 | 0.524 | **0.754** | **0.754** | **0.755** | 0.783 | 0.783 | 0.783 |
| Family information | 0.697 | 0.697 | 0.697 | 0.706 | 0.706 | 0.706 | 0.559 | 0.559 | 0.559 | **0.745** | **0.745** | **0.745** |
| Complaint/symptoms | 0.675 | 0.675 | 0.675 | **0.706** | **0.706** | **0.706** | 0.586 | 0.585 | 0.588 | 0.688 | 0.688 | 0.688 |
| Lung sounds | 0.678 | 0.678 | 0.678 | 0.561 | 0.561 | 0.561 | 0.534 | 0.534 | 0.534 | **0.682** | **0.682** | **0.682** |
| Mental state | **1.000** | **1.000** | **1.000** | **1.000** | **1.000** | **1.000** | **1.000** | **1.000** | **1.000** | **1.000** | **1.000** | **1.000** |
| Trauma | 0.757 | 0.750 | 0.766 | **0.770** | **0.770** | **0.769** | 0.647 | 0.643 | 0.653 | 0.638 | 0.638 | 0.638 |
| Airway | 0.607 | 0.595 | 0.631 | **0.707** | **0.702** | **0.714** | 0.321 | 0.310 | 0.345 | 0.458 | 0.446 | 0.482 |
| Allergies | **0.917** | **0.917** | **0.917** | 0.783 | 0.783 | 0.783 | 0.794 | 0.786 | 0.810 | 0.817 | 0.817 | 0.817 |
| Past medical history | **0.804** | **0.805** | **0.804** | 0.677 | 0.677 | 0.678 | 0.487 | 0.485 | 0.491 | 0.752 | 0.752 | 0.752 |
| AVPU | **0.683** | **0.680** | **0.686** | 0.630 | 0.628 | 0.631 | 0.623 | 0.622 | 0.624 | 0.684 | 0.684 | 0.684 |
| BGL | **0.813** | **0.813** | **0.813** | 0.672 | 0.674 | 0.670 | 0.757 | 0.759 | 0.756 | 0.677 | 0.678 | 0.676 |
| BP | **0.585** | **0.585** | **0.585** | 0.575 | 0.573 | 0.578 | 0.584 | 0.584 | 0.584 | 0.319 | 0.319 | 0.319 |
| Capillary refill time | 0.700 | 0.700 | 0.700 | **0.867** | **0.867** | **0.867** | 0.733 | 0.733 | 0.733 | 0.667 | 0.667 | 0.667 |
| ECG | **0.768** | **0.767** | **0.768** | 0.640 | 0.640 | 0.640 | 0.460 | 0.460 | 0.460 | 0.644 | 0.644 | 0.644 |
| Electrolytes | **1.000** | **1.000** | **1.000** | **1.000** | **1.000** | **1.000** | **1.000** | **1.000** | **1.000** | **1.000** | **1.000** | **1.000** |
| Pupils | 0.552 | 0.552 | 0.552 | 0.461 | 0.461 | 0.461 | **0.591** | **0.591** | **0.591** | 0.578 | 0.578 | 0.578 |
| Pulse | **0.774** | **0.774** | **0.774** | 0.546 | 0.546 | 0.546 | 0.598 | 0.598 | 0.598 | 0.665 | 0.665 | 0.665 |
| RESP | **0.716** | **0.714** | **0.719** | 0.535 | 0.535 | 0.535 | 0.646 | 0.645 | 0.648 | 0.681 | 0.679 | 0.683 |
| SPO2 | **0.655** | **0.655** | **0.655** | 0.493 | 0.493 | 0.493 | 0.604 | 0.604 | 0.604 | 0.592 | 0.592 | 0.592 |
| Temperature | **0.738** | **0.738** | **0.738** | 0.662 | 0.662 | 0.662 | 0.555 | 0.555 | 0.555 | 0.732 | 0.732 | 0.732 |
| Medication | **0.649** | **0.649** | **0.650** | 0.566 | 0.568 | 0.565 | 0.474 | 0.474 | 0.475 | 0.548 | 0.548 | 0.550 |
| Treatment | **0.576** | **0.576** | **0.577** | 0.547 | 0.548 | 0.548 | 0.486 | 0.486 | 0.487 | 0.520 | 0.520 | 0.520 |
| **Average** | **0.728** | **0.727** | **0.729** | 0.668 | 0.667 | 0.668 | 0.643 | 0.642 | 0.646 | 0.688 | 0.688 | 0.690 |

Table 4 shows the performance comparison based on the semantic similarity evaluation. Google ASR still gained the highest average performance. The performance of Google ASR in categories such as “pupils”, “past medical history,” “ECG,” “medication,” and “treatment” showed greater improvement compared to the other categories when semantic similarity evaluation was used. Through our investigation, we discovered that semantic measurement could identify cases where transcribed words have similar meanings to the ground truth, even if the words are not an exact match. For example, if the ground truth is “diarrhea for the past two days”, and the transcribed word is “diarrhea for the past few days”, the semantic similarity between these two phrases is more than 0.9, and it is counted as a correct case. On the other hand, the performance in the “temperature” category dropped slightly when the semantic similarity measurement was used because we observed that the transcribed temperature sometimes omitted the decimal point in the value.

Table 4
**Performance comparison of the ASR using semantic similarity-based accuracy (similarity > = 0.8)**. *Note*: The numbers in bold highlight the best performance of each category.

| EMS EHR fields | Google ASR | OpenAI ASR | Amazon ASR | Azure ASR |
|---|---|---|---|---|
| Gender | 0.703 | 0.739 | **1.000** | 0.962 |
| Age | 0.773 | 0.538 | 0.764 | **0.795** |
| Family information | 0.743 | 0.761 | 0.590 | **0.800** |
| Complaint/symptoms | 0.704 | **0.738** | 0.610 | 0.721 |
| Lung sounds | **0.701** | 0.582 | 0.529 | 0.662 |
| Mental state | **1.000** | **1.000** | **1.000** | **1.000** |
| Trauma | 0.761 | **0.793** | 0.679 | 0.648 |
| Airway | 0.597 | **0.727** | 0.357 | 0.489 |
| Allergies | **0.917** | 0.820 | 0.784 | 0.828 |
| Past medical history | **0.848** | 0.710 | 0.522 | 0.765 |
| AVPU | **0.713** | 0.653 | 0.641 | 0.710 |
| BGL | **0.835** | 0.674 | 0.760 | 0.696 |
| BP | **0.675** | 0.553 | 0.640 | 0.574 |
| Capillary refill time | 0.714 | **0.898** | 0.756 | 0.697 |
| ECG | **0.831** | 0.660 | 0.592 | 0.712 |
| Electrolytes | **1.000** | **1.000** | **1.000** | **1.000** |
| Pupils | **0.648** | 0.495 | 0.598 | 0.584 |
| Pulse | **0.779** | 0.562 | 0.609 | 0.687 |
| RESP | **0.753** | 0.564 | 0.658 | 0.701 |
| SPO2 | **0.684** | 0.528 | 0.640 | 0.612 |
| Temperature | 0.696 | 0.622 | 0.571 | **0.704** |
| Medication | **0.670** | 0.567 | 0.505 | 0.578 |
| Treatment | **0.619** | 0.579 | 0.527 | 0.574 |
| **Average** | **0.755** | 0.685 | 0.667 | 0.717 |

### 3.3 Types of errors in transcribing

Given that the ASRs did not perform well on some categories, we analyzed the errors and grouped them into three types: substitution (the transcribed word is different from the ground truth), approximation (the transcribed word differs by a few characters from the ground truth, but has a similar pronunciation), and truncation (the transcribed word omits certain characters from the ground truth). Table 5 exhibits some representative examples of the substitution, approximation, and truncation errors. The first five cases show substitution errors where the transcribed word(s) differ from the ground truth conversation. In other words, most of them change the meaning of the original words, which could result in wrong documentation in the EHR. The next four cases are examples of approximation errors, indicating that the ASR transcribed the words into ones that sound similar but have totally different meanings. In the medical domain, transcribing “hypertension” as “hypotension” or “hypoglycemia” as “hyperglycemia” would raise critical concerns. In the truncation error type, as demonstrated in the last three cases, instances of non-exact matches, even when only one character is omitted in the transcription, could potentially lead to a completely different representation of the patient’s situation. For instance, the acronym “OPA” stands for oropharyngeal airway, but the transcribed phrase “OP” omits the character “A”, making it inaccurately represent the patient’s status.

Table 5
Examples of the transcribing errors in each type

| Error type | Category | Ground truth conversation | Transcribed text |
|---|---|---|---|
| Substitution | AVPU | responsive | responding |
| | AVPU | crying | trying |
| | Complaint/Symptoms | cyanosis | diagnosis |
| | Medication | clonodine | klonopin |
| | Treatments | glucometer | glucose |
| Approximation | SPO2 | capnography | catnography |
| | Medication | catapres | cataphras |
| | Complaint/Symptoms | hypertension<br>syncope, bradycardia | hypotension<br>syncope, bradycardia |
| | Complaint/Symptoms | hypoglycemia | hyperglycemia |
| Truncation | Complaint/Symptoms | asystolic period | systolic period |
| | Treatments | cardiovert | cardio |
| | Treatments | OPA | OP |

### 3.4 ASR efficiency comparison

The average length of the audio files is 660 seconds. We assessed the efficiency of different ASR engines in transcribing these audio files. The average processing times for individual audio files using Google ASR, OpenAI ASR, Amazon ASR, and Azure ASR were 211.39, 265.05, 32.38, and 331.21 seconds, respectively. Notably, Amazon ASR demonstrated significantly faster performance compared to the other three services. This could potentially be attributed to its more efficient batch processing capabilities.

### 4. Discussion

With the growing interest in leveraging ASR engines to facilitate and automate clinical documentation, a few studies have examined and evaluated their performance in recognizing medical information and automating (part of) clinical documentation during patient-provider encounters. For example, Tran et al. [11] assessed the performance of two ASR engines (Google Speech-to-Text Clinical Conversation and Amazon Transcribe Medical) in recognizing non-lexical conversational sounds (e.g., “Mm-hm,” “Uh-uh”) during primary care encounters. In a similar vein, a few other studies [7] have used and evaluated ASR engines for transcribing clinical conversations in domains such as primary care [8], orthopedic encounters [27], home hemodialysis [28], and telemedicine [29]. However, these prior works were primarily conducted in quieter medical settings, such as patient exam rooms, and focused on transcribing and summarizing one-on-one patient-clinician interactions. The performance of ASR engines in more dynamic, noisy, and fast-paced environments involving conversations among several people has been understudied. To the best of our knowledge, this study is the first to examine commercially available ASR engines in transcribing clinical conversations in dynamic emergency medicine settings.

Our evaluation results show that the overall quality of the transcripts generated by current state-of-the-art ASR engines fall short in accurately transcribing and recognizing medical information in the busy and noisy EMS environment. Two of them, OpenAI Speech-to-Text and Amazon Transcribe Medical with the conversation model, performed worse than the other two in recognizing information in some critical EMS EHR fields, such as “airway” and “pupils”. For all four ASR engines, a number of pieces of content containing clinically relevant information were truncated, with many being replaced by irrelevant words, particularly within categories such as “complaint/symptoms”, “treatment”, and “medication”. Our examination of the 40 EMS audio recordings indicates that over 87.5% of communications during EMS scenes contain clinical information within these categories. Accurate documentation in these fields is therefore essential, especially for downstream tasks like generating real-time decision support to enhance EMS care. Failure to capture such information correctly could lead to inaccuracies in clinical documentation and potentially adverse patient safety incidents.

The results of this study indicate that there is substantial potential for improvement of state-of-the-art ASR engines in accurately recognizing clinical information in EMS communication, especially with the advance of AI and advanced large language models (LLMs), such as GPT-4 [30], Me-Llama [31], and others. By investigating the recordings, we are confident that some of the words can be reliably determined by a human listener based on the context of the communication. This suggests that training LLMs to correct the transcribing errors introduced by ASRs should be technically possible. Our future strategy involves creating a training dataset that encompasses the common errors made by ASR engines in the EMS context. This will enable us to fine-tune the LLMs to identify errors based on context, ensuring not only accurate transcription but also capturing the intended words and conveyed meaning effectively. Additionally, our future work also includes field testing to assess the performance of fine-tuned LLMs in transcribing real-time clinical conversations in dynamic and noisy environments such as EMS.

However, despite the great potential of ASR engines and LLMs in transcribing clinical conversations and automating EHR documentation, there are several considerations for fully implementing such technology in real clinical settings. For example, the accuracy of current ASR engines in transcribing spontaneous speech and extracting relevant clinical information from fragmented conversations has been a concern for many healthcare clinicians [32, 33]. Therefore, human validation is still required in the process of automated EMS clinical documentation to prevent any errors or biases that may arise from the system or LLMs [34–36]. Another consideration is related to user acceptance and usability of such tools; that is, whether these tools are perceived to be easy to use and useful, and how adopting them can impact clinicians’ workflow. Limited work has engaged end-users in evaluating ASR engines, with only two exceptions where a qualitative evaluation with end-users was conducted to elicit clinicians’ experience with using such tools [28, 29]. Future work should take a human-centered approach to systematically evaluate the tools’ usability, clinical validity, and impact on clinical workflow. Finally, as some medical settings (e.g., EMS) do not have a dedicated role for documentation and require clinicians to be hands-on almost all the time [12], it is critical to consider how to facilitate the use of ASR tools in such dynamic and hands-busy environment. Recent work has proposed using wearable devices (e.g., smart glasses [37]) to enable hands-free clinical documentation. Future work can explore the potential of combining ASR engines with hands-free, wearable devices integrated with EHR systems to better support real-time transcription and documentation in dynamic and hands-busy medical settings.

There are several limitations in this study. First, only 40 audio recordings from EMS training simulations were utilized to assess the ASR engines. The relatively limited sample size might affect the generalizability of our findings. Second, our results might not accurately reflect the actual performance of ASR systems when applied to real EMS environments, which are subject to various complicated factors, including possible more interruptions, variations in communications between patients and clinicians, and the potential for increased outdoor noise levels, and operating in a moving vehicle. These conditions are expected to present greater challenges and may lead to lower ASR performance. Third, our study only evaluated four ASR engines. We acknowledge the existence of other initiatives led by academic institutions and companies (such as tecdoc.ai) aimed at developing next-generation ASR engines tailored for clinical documentation. However, some ASR engines did not provide an open API for evaluation. Finally, our assessment focused on evaluating the transcription of conversations only in the EMS context. More study is needed in other noisy and dynamic settings such as emergency department and trauma resuscitations where there are more complex teams present.

### 5. Conclusion

We assessed the performance of four contemporary ASR engines in analyzing conversations within EMS or prehospital care to determine the potential of using such technology to facilitate and even automate real-time clinical documentation. Our results indicate that the Google ASR engine outperformed the other three ASR engines in recognizing clinical information across most categories in EMS EHR. However, the overall performance of all engines remains suboptimal, with significant clinical information often omitted or replaced with irrelevant words. Such errors can result in missing or incorrectly interpreted patient information and treatment during prehospital care. Therefore, there is a need to improve the performance of ASR engines to ensure accurate recognition of all critical clinical information, enabling effective automated documentation in the EMS setting. Future work should focus on enhancing ASR accuracy to minimize recognition errors and reduce patient safety risks associated with EMS clinical documentation technology.

### Declarations

## Competing Interests

Dr. Zhan Zhang is an Associate Editor of the Journal of Health Informatics Research. Other authors declare that they have no conflicts of interest.

## Ethical Approval:

Not applicable

## Funding:

This work was supported in part by funding from National Science Foundation (Award# 2237097) and National Institute of Health (Award# 1R15LM014556-01).

## Author Contribution

XL, KA, and ZZ conceptualized and designed the study. KA and ZZ provided data resources. XL and ZL conducted data analysis. XL, ZL, and ZZ wrote the main manuscript text. KA reviewed and revised the manuscript.

## Acknowledgement

We want to thank Colorado EMS for Children which supported the original data collection during simulations.

## Data Availability Statements:

Upon acceptance, the ground truth transcripts and annotated data will be deposited in a publicly available repository (e.g., Dryad Digital Repository).

## References

1. Arndt BG, Beasley JW, Watkinson MD, Temte JL, Tuan W-J, Sinsky CA (2017) Gilchrist. Tethered to the EHR: primary care physician workload assessment using EHR event log data and time-motion observations. Annals Family Med 15:419–426
2. Sinsky C, Colligan L, Li L, Prgomet M, Reynolds S, Goeders L, Westbrook J, Tutty M, Blike G (2016) Allocation of physician time in ambulatory practice: a time and motion study in 4 specialties. Ann Intern Med 165:753–760
3. Pelland KD, Baier RR, Gardner RL (2017) It is like texting at the dinner table': a qualitative analysis of the impact of electronic health records on patient–physician interaction in hospitals. BMJ Health Care Inf 24:2
4. Sarcevic A, Ferraro N (2017) On the use of electronic documentation systems in fast-paced, time-critical medical settings. Interact Comput 29:203–219
5. Park SY, Chen Y (2012) Adaptation as design: learning from an EMR deployment study. In *Proceedings of the SIGCHI Conference on Human Factors in Computing Systems*, 2097–2106
6. Park SY, Lee SY, Chen Y (2012) The effects of EMR deployment on doctors' work practices: A qualitative study in the emergency department of a teaching hospital. Int J Med Informatics 81(3):204–217

7. van Buchem MM, Boosman H, Bauer MP, Kant IM, Cammel SA (2021) Steyerberg. The digital scribe in clinical practice: a scoping review and research agenda. NPJ Digit Med 4:1
8. Shafran I, Du N, Tran L, Perry A, Keyes L, Knichel M, Domin A, Huang L, Chen Y-h, Li G (2020) The Medical Scribe: Corpus Development and Model Performance Analyses. In *Proceedings of the Twelfth Language Resources and Evaluation Conference*, 2036–2044
9. Nuance Announces the General Availability of Ambient Clinical Intelligence https://news.nuance.com/2020-02-24-Nuance-Announces-the-General-Availability-of-Ambient-Clinical-Intelligence
10. Amazon Comprehend Medical https://aws.amazon.com/comprehend/medical/
11. Tran BD, Latif K, Reynolds TL, Park J, Elston Lafata J, Tai-Seale M, Zheng K (2023) Mm-hm,Uh-uh: are non-lexical conversational sounds deal breakers for the ambient clinical documentation technology? J Am Med Inform Assoc 30:703–711
12. Zhang Z, Joy K, Harris R, Park SY (2022) Characteristics and challenges of clinical documentation in self-organized fast-paced medical work. *Proceedings of the ACM on Human-Computer Interaction*, 6, CSCW2 1–21
13. Pilerot O (2019) and H. Maurin Söderholm. A conceptual framework for investigating documentary practices in prehospital emergency care
14. Google Speech to Text https://cloud.google.com/speech-to-text?hl=en
15. Introducing Whisper - OpenAI https://openai.com/index/whisper
16. Azure Speech to Text https://azure.microsoft.com/en-us/products/ai-services/speech-to-text
17. Zhang Z, Joy K, Upadhyayula P, Ozkaynak M, Harris R, Adelgais K (2021) Data work and decision making in emergency medical services: a distributed cognition perspective. *Proceedings of the ACM on Human-Computer Interaction*, 5, CSCW2 1–32
18. Ozkaynak M, Dolen C, Dollin Y, Rappaport K, Adelgais K (2020) Simulating teamwork for better decision making in pediatric emergency medical services. In *AMIA Annual Symposium Proceedings*. American Medical Informatics Association, 993
19. NEMSIS Data Dictionary EMS Data Standard https://nemsis.org/media/nemsis_v3/release-3.5.0/DataDictionary/PDFHTML/EMSDEMSTATE/index.html
20. Yujian L, Bo L (2007) A normalized Levenshtein distance metric. IEEE Trans Pattern Anal Mach Intell 29:1091–1095
21. Şen Z (2020) Fuzzy string matching procedure. Open Bioinf J 13:1
22. H. R. Bosker. Using fuzzy string matching for automated assessment of listener transcripts in speech intelligibility studies. Behav Res Methods, 53, 5 (2021) 1945–1953
23. Reimers N, Gurevych I (2019) Sentence-BERT: Sentence Embeddings using Siamese BERT-Networks. In *Proceedings of the 2019 Conference on Empirical Methods in Natural Language Processing and the 9th International Joint Conference on Natural Language Processing (EMNLP-IJCNLP)*, 3982–3992
24. Wu Y, Jiang M, Xu J, Zhi D, Xu H (2017) Clinical named entity recognition using deep learning models. In *AMIA annual symposium proceedings*. American Medical Informatics Association, 1812
25. Luo X, Gandhi P, Storey S, Huang K (2021) A Deep Language Model for Symptom Extraction from Clinical Text and Its Application to Extract COVID-19 symptoms from Social Media. IEEE J Biomedical Health Inf
26. Navarro DF, Ijaz K, Rezazadegan D, Rahimi-Ardabili H, Dras M, Coiera E, Berkovsky S (2023) Clinical named entity recognition and relation extraction using natural language processing of medical free text: A systematic review. Int J Med Informatics, 105122
27. Enarvi S, Amoia M, Teba MD-A, Delaney B, Diehl F, Hahn S, Harris K, McGrath L, Pan Y, Pinto J (2020) Generating medical reports from patient-doctor conversations using sequence-to-sequence models. In *Proceedings of the first workshop on natural language processing for medical conversations*, 22–30
28. Lacson RC, Barzilay R, Long WJ Automatic analysis of medical dialogue in the home hemodialysis domain: structure induction and summarization. J Biomed Inform, 39, 5 (2006), 541–555
29. Joshi A, Katariya N, Amatriain X, Kannan A (2020) Dr. Summarize: Global Summarization of Medical Dialogue by Exploiting Local Structures. In *Findings of the Association for Computational Linguistics: EMNLP 2020*, 3755–3763
30. Achiam J, Adler S, Agarwal S, Ahmad L, Akkaya I, Aleman FL, Almeida D, Altenschmidt J, Altman S, Anadkat S (2023) Gpt-4 technical report. *arXiv preprint arXiv:2303.08774*
31. Xie Q, Chen Q, Chen A, Peng C, Hu Y, Lin F, Peng X, Huang J, Zhang J (2024) and V. Keloth. Me LLaMA: Foundation Large Language Models for Medical Applications. *arXiv preprint arXiv:2402.12749*

32. Quiroz JC, Laranjo L, Kocaballi AB, Berkovsky S, Rezazadegan D (2019) Coiera. Challenges of developing a digital scribe to reduce clinical documentation burden. NPJ Digit Med 2:1
33. Lin SY, Shanafelt TD, Asch SM (2018) Reimagining clinical documentation with artificial intelligence. In *Mayo Clinic Proceedings*. Elsevier, 563–565
34. Yu P, Xu H, Hu X, Deng C (2023) Leveraging generative AI and large Language models: a Comprehensive Roadmap for Healthcare Integration. Healthcare. MDPI, p 2776
35. Ong JCL, Chang SY-H, William W, Butte AJ, Shah NH, Chew LST, Liu N, Doshi-Velez F, Lu W, Savulescu J (2024) Ethical and regulatory challenges of large language models in medicine. Lancet Digit Health 6:6
36. Gerke S, Minssen T, Cohen G (2020) Ethical and legal challenges of artificial intelligence-driven healthcare. Elsevier
37. Zhang Z, Luo X, Harris R, George S, Finkelstein J (2022) Hands-free electronic documentation in emergency care work through smart glasses. In *International Conference on Information*. Springer, 314–331

## Figures

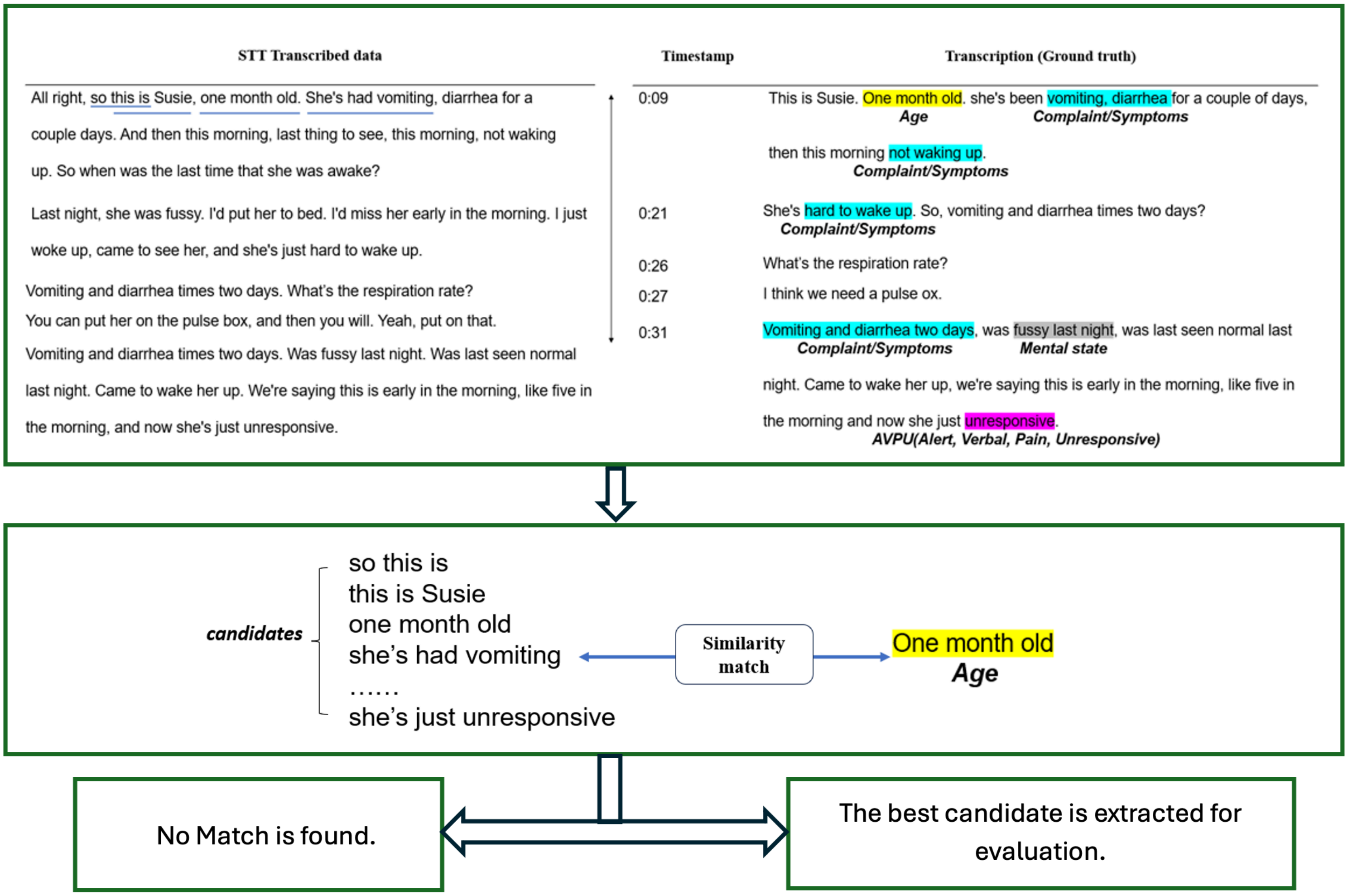

Figure 1

A text analysis framework to extract the matching content.
